# Altered vertebral and femoral bone structure in juvenile offspring of microswine subject to maternal low protein nutritional challenge

**DOI:** 10.14814/phy2.14081

**Published:** 2019-06-04

**Authors:** Stuart A. Lanham, Elizabeth DuPriest, Philipp Kupfer, Cyrus Cooper, Susan P. Bagby, Richard O. C. Oreffo

**Affiliations:** ^1^ Bone and Joint Research Group Centre for Human Development Stem Cells and Regeneration Human Development and Health Institute of Developmental Sciences Faculty of Medicine University of Southampton Southampton UK; ^2^ Division of Nephrology & Hypertension Oregon Health & Science University and Portland VA Medical Center Portland Oregon

**Keywords:** Bone, intrauterine programming, low protein, computed tomography

## Abstract

Epidemiological studies suggest skeletal growth is programmed during intrauterine and early postnatal life. We hypothesize that bone development may be altered by maternal diet and have investigated this using a microswine model of maternal protein restriction (MPR). Mothers were fed a control diet (14% protein) or isocaloric low (1%) protein diet during late pregnancy and for 2 weeks postnatally. Offspring were weaned at 4 weeks of age to ad lib or calorie‐restricted food intake groups. Femur and vertebra were analysed by micro computed tomography in offspring 3–5 months of age. Caloric restriction from 4 weeks of age, designed to prevent catch‐up growth, showed no significant effects on bone structure in the offspring from either maternal dietary group. A maternal low protein diet altered trabecular number in the proximal femur and vertebra in juvenile offspring. Cortical bone was unaffected. These results further support the need to understand the key role of the nutritional environment in early development on programming of skeletal development and consequences in later life.

## Introduction

Epidemiological data has suggested that the risk of developing a broad range of chronic diseases in adulthood, including cardiovascular disease and type 2 diabetes is influenced not only by genetic and life‐style factors, but also by environmental factors present in early life. In this process, known as developmental origins of health and disease (Gluckman and Hanson 2004), maternal nutrition plays a vital role, with disease manifest when intrauterine or early postnatal malnutrition forces trade‐offs between protection of vital organs versus growth of other tissues. Developmental plasticity, the ability of an organism to alter developmental patterns depending on the environment, provides a conceptual explanation for this phenomenon (Bateson et al. 2004). If the maternal environment is severe, for example, inadequate maternal nutrition, there may be a defensive response by the fetus to limit growth, which may be both global and asymmetric, with greater impact on organs less critical for fetal survival (liver, kidney) as compared to heart and brain (Gluckman et al. 2005). It has been hypothesized that the fetal response to maternal‐fetal nutritional stress leads to a phenotypic change which provides a fitness advantage in later life, altering its physiology to better match (“predict”) an adult environment which matches that experienced in utero. Such modifications have been termed predictive adaptive responses (Gluckman et al. 2005).

Osteoporosis is characterized by low bone mass and microarchitectural deterioration, producing bone fragility and increasing susceptibility to fracture (NIH Consensus Development Panel on Osteoporosis Prevention Devlopment and Therapy 2001). The peak bone mass attained by an individual is a major factor indicative of fracture risk in later life. Peak bone mass is partly inherited, however, current genetic markers can only partially explain the variation in an individual's bone mass or fracture risk (Ralston 1998). Through epidemiological evidence, reduced bone mass in the adult is associated with impaired growth during fetal life, infancy, and early childhood (Cooper et al. 1997, 2002; Jones et al. 2000; Tobias et al. 2005). These observations indicate a link between maternal and fetal nutrition, peak bone mass, and fracture risk in later life. Cohort studies have demonstrated significant intrapair concordance between birth weight and bone mass in twins, including monozygous twins (Antoniades et al. 2003). These results suggest that other influences, distinct from genetics, can affect birth weight and bone mass, and suggest that variations in the intrauterine environment, even within the normal range, may be an additional factor. We hypothesized that bone development may be altered by maternal diet and have investigated this using a microswine model of maternal protein restriction (MPR).

While currently under investigation, the mechanisms involved in these phenotypic changes, have been conceptualized in terms of the detrimental consequences of mismatch between the postnatal environment predicted on the basis of maternal cues during development and the environment actually experienced by the offspring. Indeed the suggestion that some components of development constitute predictive adaptive responses (PAR) has received support from studies of vascular function or body fatness (Khan et al. 2004; Jasienska et al. 2006; Cleal et al. 2007).

Here we present data from a larger mammalian model, the microswine, which show altered bone structure and density in the femur and vertebra in juvenile offspring from mothers fed a low protein diet during the last quarter of pregnancy and the early postnatal period, during which maximal bone development occurs.

## Methods and Materials

### Ethical approval

Sows were housed at the Oregon Health & Science University (OHSU) Department of Comparative Medicine and experiments were approved by the OHSU Institutional Animal Care and Use Committee under protocol A439, under compliance with the Animal Welfare Act regulations and Public Health Service (PHS) Policy.

### Experimental design and animal care

Time‐mated microswine sows were obtained from Sinclair Research Laboratories (formerly Charles River Laboratories) approximately two‐thirds into gestation. Sows were maintained on a 12 h:12 h light:dark cycle in a temperature‐controlled room. Cages were placed in the same room adjacent to each other to allow pigs to have social contact with each other. From gestation day 84 (term is gestational day 115), sows were randomly assigned to normal or isocaloric low protein (1%, custom formulated) diet (DuPriest et al. 2012b). The duration of protein restriction was designed to approximate the last trimester of human gestation in terms of renal development, and, critically, also approximates a similar period for bone development. As renal and bone tissue development is somewhat delayed in microswine vs. humans, sows were returned to a normal diet (Purina Mills Lab Porcine Diet Grower) at 2 weeks after delivery. Sows and offspring were housed under 12 h:12 h light:dark cycle at constant temperature, and ceramic heaters were available from 2 days prior to the expected due date through weaning for piglets. At ~4 weeks of age, piglets were weaned to ad libitum or calorie‐restricted diets (see below). Data from these animals have been previously published (DuPriest et al. 2012a,b, 2018).

### Caloric restriction

It was previously reported that body weight reduction in low protein offspring was greater at 2 weeks of age than at birth (DuPriest et al. 2012b). The weight deficit at 2 weeks for each animal was therefore calculated as a percentage of the sex‐matched control protein offspring average. Calorie‐restricted offspring were offered a specific amount of feed designed to keep offspring weights reduced to the same degree throughout postnatal development as determined at 2 weeks of age for each individual as previously described (DuPriest et al. 2018). Given low protein offspring were previously shown to eat ~16% more feed than control protein offspring, feed was initially offered at 25 g/kg/meal (two meals per day), approximately the same amount consumed by control protein offspring. However, as low protein offspring also exhibited increased feed utilization efficiency, the amount of feed offered was adjusted as necessary every 2–3 days, never falling below 20 g/kg, to maintain calorie restricted offspring on the projected growth trajectory (DuPriest et al. 2018). Thus the degree of caloric restriction averaged 20–25% of control dietary intake. Average feed intake (g/kg) across the period of caloric restriction did not differ between low protein and normal protein offspring (DuPriest et al. 2018).

### Micro computed tomography

Femora and vertebrae from offspring were scanned using an Xtek Benchtop 160Xi scanner (Nikon Metrology, Tring, Hertfordshire, UK) equipped with a Hamamatsu C7943 x‐ray flat panel sensor (Hamamatsu Photonics, Welwyn Garden City, Hertfordshire, UK). All scans were taken at 100 kV, 60 μA using a molybdenum target with an exposure time of 534 ms and 4× digital gain. Femoral samples were scanned at 50 μm resolution and L2 vertebral samples scanned at 67 μm resolution. Reconstructed volume images were analyzed using VGStudio Max 1.2.1 software (Volume Graphics GmbH, Heidelberg, Germany). All the voxels which formed the structure were automatically assigned Hounsfield units. Additional calculations of structural model index (SMI) and trabecular pattern factor were performed using a custom written package and the Visilog Quantification + package (both Noesis, Crolles, France) within the Amira 4.1.2 package (Mercury Computer System Inc., Chelmsford, USA). SMI is a method for determining the plate‐ or rod‐like geometry of trabecular bone with values of 0 for plates and 3 for rods. Trabecular pattern factor (TbPf) is an inverse index of connectivity, hence, a lower (closer to zero) TbPf signifies improved connected trabecular lattices while a higher (further from zero) TbPf indicates a more disconnected trabecular structure.

### Dual‐energy x‐ray absorptiometry

At 6 weeks (when weights were most reduced in low protein offspring vs. control offspring in pilot studies) and 11 weeks (when weights were statistically similar between low protein offspring and control offspring in pilot studies), bone mineral content and bone mineral density were assessed by dual‐energy x‐ray absorptiometry (DEXA) (DuPriest et al. 2012b, 2018). Piglets were anesthetized by inhaled isoflurane (2.0–2.5% isoflurane, up to 3.0% during the first minutes of induction, with an oxygen flow rate of 2.0 L/min) and placed prone on the DEXA scanner (Hologic QDR‐4500W). Forelimbs were excluded due to anatomic constraints imposed by use of paediatric software (Experimental Paediatric Whole Body v8.26 & v12.3). DEXA data were obtained from 32 total juvenile offspring, from 3 low protein and 3 normal protein sows. All healthy offspring were studied. However, some animal losses occurred over the course of experiments.

### Tissue collection

At 14–25 weeks (i.e., 3–5 months), juvenile offspring were placed under isoflurane anesthesia and animals were sacrificed by removal of vital organs, including the heart (DuPriest et al. 2018). Following removal of vital organs, one femur and three lumbar vertebrae (L1, L2, and L3) were dissected from each animal and stored at −80°C until analyzed. The same animals were studied as in the DEXA experiments.

### Statistics

T‐tests and Wilcoxon‐Mann–Whitney statistical analysis was used to determine the effect of maternal or postweaning diets, and two‐way ANOVA with Bonferroni correction was performed to determine the effect of sex within the diet group using the SPSS for Windows program version 23 (IBM Corp, Portsmouth, Hampshire, UK). Three‐way ANOVA with Bonferroni correction was used to determine any effect of sample age. Data presented as mean ± SD or 95% confidence limits and significance was determined with a *P*‐level of 0.05 or lower.

## Results

### Effect of combined maternal low protein and postweaning caloric restricted diets

Bone mineral content (BMC) and bone mineral density (BMD) were assessed by DEXA for total body (excluding head and forelimbs), right hind leg and lumbar vertebrae on the four diet groups (Table 1). The maternal low protein diet alone resulted in offspring with lower total BMC, lower hind leg and vertebrae BMC and raised total BMD at 6 weeks of age, and raised vertebral BMC at 11 weeks of age (all *P* values < 0.05). The only observed effect of postweaning caloric restriction on control animals was a lower vertebral BMC at 6 weeks of age. Postweaning caloric restriction on the maternal low protein group prevented the increase in total BMD at 6 weeks of age.

**Table 1:** Bone mineral content and density in offspring at 6 and 11 weeks of age

	Maternal + 2 weeks postnatal Diet			
	Control Mean (SD)		Low Protein Mean (SD)	
	Diet from Weaning		Diet from Weaning	
	Ad libitum	Restricted	Ad libitum	Restricted
	*n* = 6	*n* = 12	*n* = 8	*n* = 6
Body Mass at 6 weeks (kg)	6.3 (0.8)^a^	5.4 (1.2)^ab^	4.9 (0.4)^ab^	4.4 (0.8)^b^
Body Mass at 11 weeks (kg)	18.8 (1.0)^ac^	15.2 (2.3)^bc^	16.0 (3.0)^ac^	11.9 (3.1)^b^
Total BMC 6 weeks (g)	86 (20)^a^	63 (24)^ab^	50 (5)^b^	41 (11)^b^
Total BMC 11 weeks (g)	210 (23)	184 (29)	206 (40)	158 (39)
Total BMD 6 weeks (gcm^−2^)	0.34 (0.04)^a^	0.35 (0.03)^a^	0.40 (0.02)^b^	0.37 (0.01)^a^
Total BMD 11 weeks (gcm^−2^)	0.52 (0.03)	0.50 (0.02)	0.53 (0.05)	0.49 (0.06)
Right Leg BMC 6 weeks (g)	15 (3)^a^	11 (5)^ab^	7 (1)^b^	7 (2)^b^
Right Leg BMC 11 weeks (g)	38 (4)^a^	33 (6)^ab^	33 (6)^ab^	25 (8)^b^
Right Leg BMD 6 weeks (gcm^−2^)	0.30 (0.03)	0.30 (0.03)	0.31 (0.02)	0.30 (0.02)
Right Leg BMD 11 weeks (gcm^−2^)	0.53 (0.04)^a^	0.50 (0.03)^ab^	0.50 (0.06)^ab^	0.44 (0.07)^b^
Lumbar Vert BMC 6 weeks (g)	6 (2)^a^	4 (1)^b^	3 (1)^b^	4 (1)^b^
Lumbar Vert BMC 11 weeks (g)	15 (4)^ab^	14 (4)^a^	19 (4)^b^	15 (2)^ab^
Lumbar Vert BMD 6 weeks (gcm^−2^)	0.42 (0.07)	0.41 (0.04)	0.41 (0.03)	0.42 (0.02)
Lumbar Vert BMD 11 weeks (gcm^−2^)	0.54 (0.07)	0.50 (0.04)	0.52 (0.04)	0.49 (0.03)

BMC and BMD measurement for whole body (excluding head and forelimbs), right hind leg and lumbar vertebrae are shown as measured at 6 and 11 weeks of age in offspring from mothers fed either control or low protein diet during the last quarter of pregnancy and first 2 weeks of postnatal life. Caloric restriction was from weaning at 4 weeks of age and was designed to keep offspring weights reduced to the same degree throughout postnatal development as determined at 2 weeks of age for each individual. All values shown are mean (standard deviation). For each parameter measured, values with different superscript letters are significantly different from each other (*P* < 0.05).

Femur and lumbar vertebra analyses in the four experimental groups are shown in Table 2. Differences were only observed in the lumbar vertebra. The maternal low protein diet reduced structural model index in Ad Libitum fed animals (both *P* values < 0.05). The postweaning caloric restriction produced a lower trabecular spacing (*P* value < 0.05). Sample age did not affect the results of these analyses.

**Table 2:** Distal femur and vertebral bone structure in offspring at 3–5 months of age

	Maternal + 2 weeks postnatal Diet			
	Control Mean (SD)		Low Protein Mean (SD)	
	Diet from Weaning		Diet from Weaning	
	Ad libitum	Restricted	Ad libitum	Restricted
	*n* = 6	*n* = 11	*n* = 8	*n* = 7
Femur mass (g)	31 (7)	32 (8)	31 (9)	30 (6)
Femur length (mm)	133 (12)	137 (10)	131 (15)	133 (11)
Proximal Femur				
Bone Volume (cm^3^)	8.8 (1.6)	8.9 (2.4)	8.8 (2.3)	9.4 (1.3)
BS/BV	10.0 (1.5)	9.3 (1.3)	10.6 (1.0)	10.2 (0.8)
BV/TV	0.55 (0.04)	0.57 (0.04)	0.56 (0.03)	0.56 (0.03)
Trabecular Thickness (mm)	0.20 (0.03)	0.22 (0.03)	0.19 (0.02)	0.20 (0.02)
Trabecular Spacing (mm)	0.16 (0.03)	0.16 (0.01)	0.15 (0.02)	0.16 (0.01)
Trabecular Number per mm	2.8 (0.4)	2.7 (0.3)	3.0 (0.3)	2.8 (0.1)
Structural Model Index	0.64 (0.15)	0.60 (0.09)	0.53 (0.06)	0.60 (0.06)
Trabecular Pattern Factor	−10.5 (0.9)	−9.6 (0.6)	−10.1 (1.3)	−10.1 (1.2)
Femoral midshaft				
Cortical Thickness (mm)	3.3 (0.2)	3.1 (0.3)	3.3 (0.4)	3.3 (0.5)
Diameter (mm)	15.0 (1.6)	15.8 (2.0)	15.3 (2.2)	15.0 (2.6)
Lumen area (mm^2^)	58 (23)	74 (30)	63 (30)	60 (34)
Cross‐sectional area (mm^2^)	180 (39)	199 (50)	186 (52)	181 (62)
Cross Sectional Moment of Inertia (mm^4^)	2400 (950)	2800 (1200)	2600 (1300)	2500 (1600)
Vertebra				
Bone volume (mm^3^)	820 (250)	880 (230)	800 (260)	840 (230)
BS/BV	10.2 (3.4)	9.5 (1.5)	9.9 (1.6)	10.2 (1.8)
BV/TV	0.51 (0.04)	0.53 (0.03)	0.55 (0.05)	0.55 (0.01)
Trabecular Thickness (mm)	0.24 (0.03)	0.22 (0.04)	0.21 (0.03)	0.20 (0.03)
Trabecular Spacing (mm)	0.13 (0.07)	0.19 (0.03)	0.17 (0.01)	0.16 (0.03)
Trabecular Number per mm	2.2 (0.4)	2.5 (0.4)	2.7 (0.2)	2.8 (0.5)
Structural Model Index	0.68 (0.12)^a^	0.49 (0.08)^ab^	0.46 (0.05)^b^	0.45 (0.17)^b^
Trabecular Pattern Factor	−6.3 (0.7)	−5.5 (1.4)	−6.6 (1.0)	−6.1 (0.3)

Structural data shown are for femur and L2 lumbar vertebra from offspring from offspring from mothers fed either control or low protein diet during the last quarter of pregnancy and first 2 weeks of postnatal life. Caloric restriction was from weaning at 4 weeks of age and was designed to keep offspring weights reduced to the same degree throughout postnatal development as determined at 2 weeks of age for each individual. All values shown are mean (standard deviation). For each parameter measured, values with different superscript letters are significantly different from each other (*P* < 0.05).

### Effect of maternal low protein diet alone

The effect of the maternal diet was examined, irrespective of the postweaning calorie restriction. At 6 weeks of age, the low protein offspring group displayed lower mass, total BMC, and right hind leg BMC, but increased total body BMD (all *P* values < 0.011). At 11 weeks of age, the same animals displayed lower mass and hind leg BMC, but increased vertebral BMC (all *P* values < 0.03).

**Figure 1:**
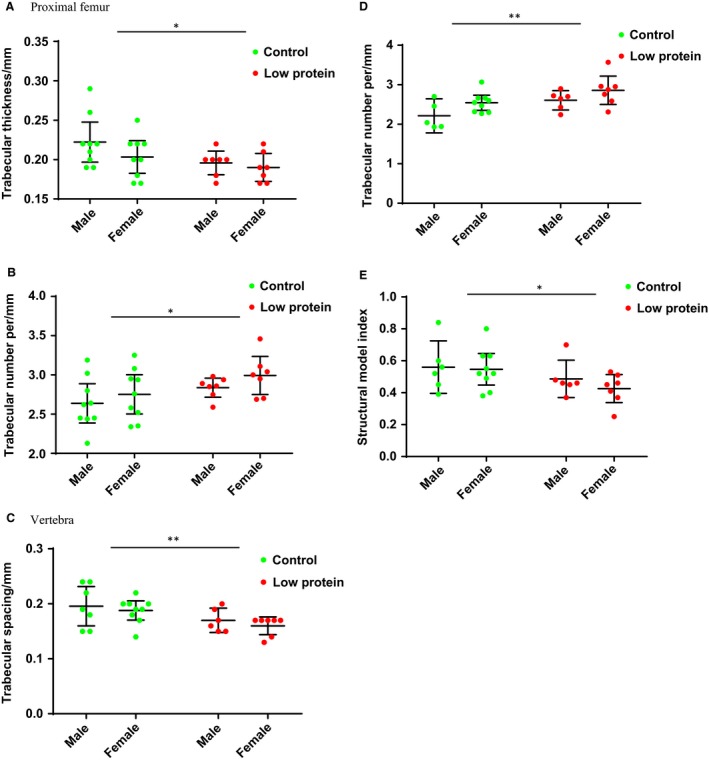
Maternal diet ‐ CT analysis of femur at 3 to 5 months of age. Ad libitum and calorie‐restricted groups shown in Table 2 were combined for each maternal diet group to determine any overall effect of the maternal diet regardless of postweaning diet. For the proximal femur, plots are shown by sex for (A). Trabecular thickness, (B). Trabecular number per mm. For the lumbar vertebra, plots are shown for (C). Trabecular spacing, (D). Trabecular number per mm, and (E). Structural model index. *P*‐values are * <0.05 and ** <0.01. Graphs show mean plus 95% confidence limits.

At 3–5 months of age, for the proximal femur, compared to controls, the maternal low protein diet produced increased bone surface to bone volume ratio (BS/BV, 10.4 ± 0.7 vs. 9.5 ± 1.9, *P* value = 0.04), reduced trabecular thickness (0.19 ± 0.00 mm vs. 0.21 ± 0.00 mm, *P* value = 0.05, Fig. 1A), and increased trabecular number per mm (2.91 ± 0.04 vs. 2.68 ± 0.11, *P* value = 0.03, Fig. 1B). The maternal low protein diet did not affect the total femur length in the offspring. Mean mass was observed to be the same in each maternal diet group (maternal control group 31.9 kg vs. maternal low protein group 30.6 kg, *P* value 0.8). Sample age and sex did not affect the results observed.

For the vertebra, compared to controls, the maternal low protein diet produced an increased bone volume to total volume ratio (BV/TV) (0.55 ± 0.00 vs. 0.52 ± 0.00, *P* value = 0.03), reduced trabecular spacing (0.16 ± 0.00 mm vs. 0.21 ± 0.00 mm, *P* value = 0.002, Fig. 1C), increased number of trabeculae per mm (2.77 ± 0.12 vs. 2.36 ± 0.07, *P* value = 0.003, Fig. 1D), and reduced SMI (0.45 ± 0.01 vs. 0.56 ± 0.02, *P* value = 0.012, Fig. 1E). When analysed by sex, only female offspring tended to be different; males were unaffected. Sample age did not affect the results of these analyses.

### Effect of postweaning caloric restriction alone

The effect of the postweaning caloric restriction was examined, irrespective of the maternal diet. At 6 weeks of age, no differences were found between the postweaning groups for mass, or any of the BMC or BMD measurements. At 11 weeks of age, the postweaning calorie‐restricted offspring displayed lower mass, total BMC, and total BMD (all *P* value < 0.02).

**Figure 2:**
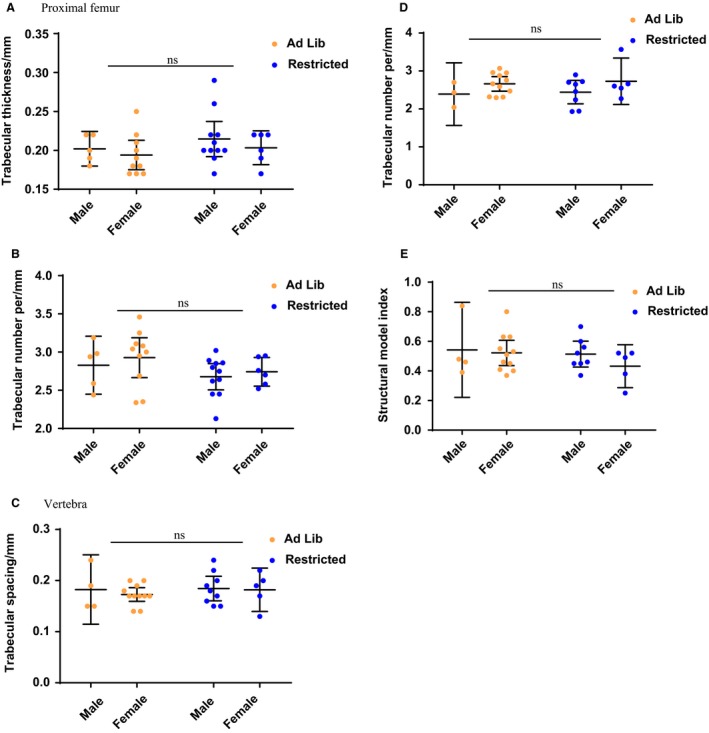
Postweaning diet ‐ CT analysis of femur at 3–5 months of age. Ad libitum results from both maternal diet groups, and calorie‐restricted results from both maternal diet groups shown in Table 2 were combined to determine any overall effect of the postweaning diet regardless of maternal diet. For the proximal femur, plots are shown by sex for (A). Trabecular thickness, (B). Trabecular number per mm. For the lumbar vertebra, plots are shown for (C). Trabecular spacing, (D). Trabecular number per mm, and (E). Structural model index. *P*‐values are ns – not significant. Graphs show mean plus 95% confidence limits.

At 3–5 months of age, the same animals displayed no significant differences in any of the parameters measured between the ad lib and restricted feed groups in either the vertebra or the proximal femur (Fig. 2A–E). Total femur length was unaffected by the postweaning caloric restriction (data not shown). Sample age and sex did not affect the results observed.

## Discussion

In these studies, at 6 weeks of age, despite lower mass and BMC, the low protein offspring samples displayed increased BMD. This suggests accelerated bone development in these programmed offspring. No differences were observed in the midshaft bone characteristics between any of the experimental groups in this study, suggesting only trabecular bone was affected by maternal or postweaning diet. However, this could simply be due to the fact that the cortical bone has a lower surface area to volume ratio than trabecular bone, and hence the action of osteoblasts and osteoclasts would be more significant on trabecular bone. If so, cortical bone may not reveal differences based on maternal diet until the offspring reaches an advanced age.

The current results illustrate the importance of the maternal diet, as the offspring from low‐protein fed mothers displayed differences in trabecular bone structure at 3–5 months of age, whereas no differences were found in the postweaning calorie‐restricted offspring. The vertebra displayed diet‐induced differences at 3–5 months of age, despite the differences in the proximal femur no longer being significant. Interestingly, the differences seen in the vertebra between the control and low protein offspring groups (trabecular spacing, trabecular number per mm, and SMI) when ad lib and calorie‐restricted groups were combined, showed larger differences in females than males. This is in agreement with previous studies which have shown male rat offspring are less affected than females by maternal diet (Lanham et al. 2011).

The purpose of the postweaning calorie restriction was to prevent the subsequent catch‐up growth seen when low protein diet restriction was removed. As this postweaning feed restriction had little impact on bone structure, these results suggests catch‐up growth may not directly alter bone structure, and that the differences observed are solely due to the low protein maternal diet. Nevertheless, body length was reduced by a consistent amount by calorie restriction (DuPriest et al. 2018), suggesting bone size, but not structure, was altered by calorie restriction.

The same juvenile microswine offspring have been studied previously, examining both whole‐body growth and hormonal states of the animals described in this work. (DuPriest et al. 2012b, 2018). The suggestion of accelerated bone development (see above) is in conjunction with accelerated whole‐body growth in crown‐rump length from 5 to 12 weeks of age. Furthermore, this accelerated growth and bone maturation occurs in the setting of low plasma growth hormone, but normal plasma IGF‐1 levels, suggesting hypersensitivity to both growth hormone and IGF‐1 (DuPriest et al. 2018). In addition, plasma adiponectin levels in low protein offspring were reduced compared to controls. Others have consistently shown circulating adiponectin levels to be inversely correlated with BMD (Naot et al. 2017), which we now show to be increased in low protein offspring at 6 weeks of age. It is possible the described hormonal changes were directly responsible for the changes to bone growth and development observed.

The data presented on these microswine in this study, and those previously reported, suggest that maternal diet plays a greater role in bone development and other systems, than a 20–25% postweaning calorie restriction (designed to prevent catch‐up growth). However, limiting catch‐up growth in low protein offspring, unexpectedly, did not improve the physiological measures in these animals. Rather, low protein offspring were observed to be more vulnerable to the negative effects of postweaning caloric restriction (DuPriest et al. 2018) (glucose metabolism being the notable exception). These suggest that rather than being predictive‐adaptive responses, the changes observed in these nutritionally programmed microswine yield a lifelong vulnerability to adverse environmental influences. There is also increasing evidence that bone turnover, insulin, leptin, and energy metabolism are all connected (Karsenty and Ferron 2012). Hence, it may be possible that long‐term altered bone structure (through modulated osteoblast and osteoclast activity) would lead to modified hormone levels from these and other bone cells (Bonnet 2017). In turn, these hormones target other organs such as pancreas, adipose tissue, liver, and muscle; all key in energy metabolism and hence glucose metabolism. Therefore this may alter the susceptibility to diabetes and metabolic syndrome.

In conclusion, we have shown that a maternal low protein diet during late gestation affects bone structure in microswine offspring up to 5 months of age, as assessed using micro‐CT. This indicates a key role of the nutritional environment in early development and, potentially, on the programming of skeletal development with implicit consequences in later life. Current studies are centered on elucidating the mechanisms involved at the cellular level.

## Conflict of Interest

None declared.
